# Developing GIS-based eastern equine encephalitis vector-host models in Tuskegee, Alabama

**DOI:** 10.1186/1476-072X-9-12

**Published:** 2010-02-24

**Authors:** Benjamin G Jacob, Nathan D Burkett-Cadena, Jeffrey C Luvall, Sarah H Parcak, Christopher JW McClure, Laura K Estep, Geoffrey E Hill, Eddie W Cupp, Robert J Novak, Thomas R Unnasch

**Affiliations:** 1 School of Medicine, Department of Infectious Diseases, University of Alabama at Birmingham, 845 19th Street South, Birmingham Alabama, USA, 35294; 2 Department of Entomology and Plant Pathology, Auburn University, 301 Funchess Hall, Auburn, Alabama, USA 36849; 3 NASA -NSSTC, Global Hydrology and Climate Center, 320 Sparkman Drive, Huntsville, Alabama, USA 35805; 4 Department of Anthropology, University of Alabama at Birmingham, 1401 University BLVD, Heritage Hall Room 360, Birmingham, Alabama, USA; 5 Department of Biological Sciences, Auburn University, Room 101, Rouse Life Science Building, Auburn, Alabama USA, 36849; 6 Department of Entomology and Plant Pathology, Auburn University, 301 Funchess Hall, Auburn, Alabama, USA 36849; 7 Global Infectious Disease Research Program, Department of Public Health, College of Public Health, University of South Florida, 3720 Spectrum Blvd, Suite 304, Tampa, Florida, USA 33612

## Abstract

**Background:**

A site near Tuskegee, Alabama was examined for vector-host activities of eastern equine encephalomyelitis virus (EEEV). Land cover maps of the study site were created in ArcInfo 9.2^® ^from QuickBird data encompassing visible and near-infrared (NIR) band information (0.45 to 0.72 μm) acquired July 15, 2008. Georeferenced mosquito and bird sampling sites, and their associated land cover attributes from the study site, were overlaid onto the satellite data. SAS 9.1.4^® ^was used to explore univariate statistics and to generate regression models using the field and remote-sampled mosquito and bird data. Regression models indicated that *Culex erracticus *and Northern Cardinals were the most abundant mosquito and bird species, respectively. Spatial linear prediction models were then generated in Geostatistical Analyst Extension of ArcGIS 9.2^®^. Additionally, a model of the study site was generated, based on a Digital Elevation Model (DEM), using ArcScene extension of ArcGIS 9.2^®^.

**Results:**

For total mosquito count data, a first-order trend ordinary kriging process was fitted to the semivariogram at a partial sill of 5.041 km, nugget of 6.325 km, lag size of 7.076 km, and range of 31.43 km, using 12 lags. For total adult *Cx. erracticus *count, a first-order trend ordinary kriging process was fitted to the semivariogram at a partial sill of 5.764 km, nugget of 6.114 km, lag size of 7.472 km, and range of 32.62 km, using 12 lags. For the total bird count data, a first-order trend ordinary kriging process was fitted to the semivariogram at a partial sill of 4.998 km, nugget of 5.413 km, lag size of 7.549 km and range of 35.27 km, using 12 lags. For the Northern Cardinal count data, a first-order trend ordinary kriging process was fitted to the semivariogram at a partial sill of 6.387 km, nugget of 5.935 km, lag size of 8.549 km and a range of 41.38 km, using 12 lags. Results of the DEM analyses indicated a statistically significant inverse linear relationship between total sampled mosquito data and elevation (R^2 ^= -.4262; p < .0001), with a standard deviation (SD) of 10.46, and total sampled bird data and elevation (R^2 ^= -.5111; p < .0001), with a SD of 22.97. DEM statistics also indicated a significant inverse linear relationship between total sampled *Cx. erracticus *data and elevation (R^2 ^= -.4711; p < .0001), with a SD of 11.16, and the total sampled Northern Cardinal data and elevation (R^2 ^= -.5831; p < .0001), SD of 11.42.

**Conclusion:**

These data demonstrate that GIS/remote sensing models and spatial statistics can capture space-varying functional relationships between field-sampled mosquito and bird parameters for determining risk for EEEV transmission.

## Introduction

Eastern equine encephalitis virus (EEEV) is the most dangerous endemic arbovirus in the United States. Up to 70% of symptomatic cases in humans are fatal [1], and most survivors are permanently debilitated by neurologic sequelae [2]. Besides the endemic and economic burdens to humans, frequent equine cases and sporadic mass game bird die-offs are costly consequences of EEEV transmission [3-5]. Epornitics in wild birds are also dramatic consequences of EEEV [6], such as die-offs of the endangered whooping crane, *Grus americana *[7]. Except in Florida [8,9], the ecology of EEEV is less understood in the southeastern United States than in other endemic locations in the region. This disease is endemic in Alabama with viral activity varying between years. The summer of 2001 was a particularly active year for EEEV, with one human and over 30 veterinary cases in the central and southern regions of the state [10].

The mosquito species *Culiseta melanura *is generally believed to initiate EEEV transmission to wild birds [11,12]. Passerine birds are the major enzootic reservoirs, and early transmission among the local avifauna is believed to be initiated by ornithophilic species, such as *Cs. melanura *[11-13]. However, peaks in abundance of *Cs. melanura *species do not correlate directly with peaks in EEEV transmission [14]. Differences in sampled abundance count data suggest that multiple mosquito species are necessary as vectors to account for large epizootics [11]. In addition to *Cs. melanura*, several other mosquito species are likely involved as bridge vectors for EEEV transmission. These species include: *Aedes vexans, Coquillettidia perturbans*, *Culex erraticus*, while *Culex peccator*, *Culex territans *and *Uranotaenia sapphirina *are suspected of circulating EEEV among reptiles and amphibians [15,16]. Of these previously listed species, it is suspected that *Cx. erraticus *is the most important EEEV bridge vector between birds and mammals in the mid-south, because of frequent virus isolations and its abundance in bottomland swamps, flood plains, permanent standing water, recreation areas near rivers or ponds, and water impoundments in Alabama and throughout the Tennessee Valley [10,17,18]. Understanding the spatial distribution of this habitat-restricted species is valuable for predicting risk of EEEV infection for nearby human populations.

Despite the misnomer "equine," EEEV transmission initiates in the avian cycle. Antibody prevalence in wild birds associated with freshwater swamps in Alabama range from 6-85% [19], which suggests that different bird species vary in attractiveness to mosquitoes and defensive behaviors against mosquito bites [20]. In Macon County, Alabama, avian species overrepresented in mosquito bloodmeals included: Yellow-Crowned Night-Heron, Carolina Chickadee, Great Blue Heron, Northern Mockingbird, and Wild Turkey [21]. Therefore, determining the spatial distribution of common bloodmeal hosts of mosquito vectors is a critical step to predicting early cycles of EEEV transmission.

Predicting foci of EEEV positive mosquitoes has been difficult, perhaps as a result of movement of human and horse populations and fluctuations in bird populations over the years [9]. Spatio-temporal distribution of arboviral vectors and hosts vary over short distances, based on differences in land cover and meteorological shifts. For example, human cases of West Nile Virus (WNV) and St. Louis Encephalitis (SLE) clustered in urban/suburban areas in Georgia and Alabama [22,23]; whereas, EEEV transmission was restricted to freshwater swamps in Florida [9]. Compared to other arboviral diseases, EEEV transmission tends to be more spatially isolated [8,9], with the notable exception of the 1989 Atlantic and Gulf coast outbreaks, which caused 196 equine cases and 9 human cases [3]. Evidence for spatial isolation of EEEV foci include the lack of early warning of transmission with sentinel flocks and very low seroconversions of both sentinel flocks (2%) and human populations within EEEV foci (1.7%) [3,8,9,24], suggesting few asymptomatic cases. Therefore, untargeted or random interventions would be excessive and wasteful [25], as EEEV vectors and hosts are not randomly distributed.

Quantification of vector-host interactions, by incorporating high resolution remotely sensed data in GIS, can help predict arbovirus transmission cycles by identifying site specific environmental predictors [25-32]. For example, in earlier research, Jacob et al. [31] found that land use land cover (LULC) change sites can aid in spatial prediction of human exposure to *Culex *mosquitoes using GIS-generated models. A LULC classification, based on Landsat-7 ETM+ data acquired in July 2003 and Landsat-5 TM data acquired in July 1991, was compared to the abundance of *Culex restuans *and *Culex pipiens *egg rafts in Urbana-Champaign, Illinois. Total LULC change, from 1991 to 2003 in the Urbana-Champaign study site, was relatively low (12.1%). The most frequent LULC category was maintained urban. The urban land cover was further subdivided by degree of tree canopy coverage using QuickBird visible and near infra-red (NIR) data, which revealed 73.3% of the urban area was in the category classified as high canopy coverage, with 20% of the remotely stratified data categorized as moderate canopy coverage, and 6.7% as low coverage. The remote stratification of the urban land cover revealed that 83.3% egg raft distribution was in the high coverage areas [31].

Characteristics of drainage networks and basin physiographic parameters have also been used in hydrologic calculations and land cover modeling of flood and swamp water mosquito abundance, using satellite data [32-36]. The automated generation of drainage networks has become increasingly popular with the use of GIS and availability of digital elevation models (DEMs). These models account for topographic variability and their control over soil moisture heterogeneity and runoff within a watershed by using a flow distance to stream grid-based analyses. The advantage of using a flow distance-to-stream algorithm generated in a DEM is that landscape profiles can be evaluated and terrain covariates can be generated, which can estimate relationships between a response variable and other environmental-sampled variables [35]. Topographic derivatives generated from a DEM can also be calculated at different scales, using the linear interpolation technique built in GIS, which can accurately yield several catchment hydrological variables, including percent surface saturation and total surface runoff for identification of potential mosquito and avian sampling sites [33].

Vector-borne disease risk can also be modeled with high predictive accuracy by using geostatistical kriging algorithms in GIS. Kriging is equated with spatial optimal linear prediction, where the unknown random-process mean is estimated with the best linear unbiased estimator. Kriging field and remote-sampled mosquito and avian predictor variables require the use of various geostatistical techniques to interpolate the parameters of a random field (e.g., the elevation, *z*, of the landscape as a function of the geographic location, at an unsampled location from data at nearby sampled locations) [34]. Stochastic kriging can also be used to generate prediction of abundance and distribution data, which can allow for numerical quantification of uncertainty estimates in arboviral explanatory covariates [31]. Additionally, predicting landscape classes in urban environments can reveal local spatial patterns of the physical and socio-economic factors hypothesized to be associated with arboviral transmission. For example, in northern California, kriging interpolation revealed that *Culex tarsalis *was the most abundant species in ovitraps near agricultural sites; whereas, *Cx. pipiens *was clustered within residential areas [33].

The dynamics of transmission of any arthropod-borne infection is a complex function of many factors, which may include the intensity of infection in the vertebrate reservoir, the competence of the vector, and the degree of contact of the vector with the infected vertebrate host reservoir [37]. Thus, generating models of EEEV, using field and remote-sampled mosquito and avian data, is essential to understanding the ecology of EEEV and for developing effective means to control outbreaks. GIS/remote sensing and spatial statistics can map interactions between arthropod mosquito vectors and avian amplification host populations, which can aid in spatially targeting high density foci of mosquito and avian sampling sites [31]. Treatments or habitat perturbations should be based on the surveillance of the most productive areas of an ecosystem [25]. Therefore, the objectives of this research were: a) to generate multiple regression models to determine predictors associated with the sampled mosquito and avian data; (b) to develop spatial linear prediction models of potential avian and mosquito sampled sites; and, c) to construct a DEM to identify terrain covariates associated with sampled mosquito and bird data in Tuskegee, Alabama.

## Materials and methods

### Study Site

The study site is located in the Tuskegee National Forest in Macon County, Alabama. Since the site was abandoned in the 1900s, it has undergone extensive re-encroachment of forest over depleted farmland and is characterized by forested bottomland wetlands [10]. The center of Tuskegee, AL is located approximately 3 km from the edge of the study site, an urban center with a human population density of 3,700 persons/km^2 ^http://factfinder.census.gov. The western edge of the sampling grid abutted the City Lake, east of the center of Tuskegee, an area with a human population density of 1,100-1,600 persons/km^2^. The northwest portion of the sampling grid also overlapped with populated areas northeast of Tuskegee and north of highways US-29/AL-81, with a human population density of also 1,100-1,600 persons/km^2^. The geographic coordinates of the centroid of the sampling grid were 85.644444 by 32.432494 decimal degrees. The central and southern portions of the sampling grid had a human population density of 80 persons/km^2^, and the eastern edge had a human population density of 0-50 persons/km^2^.

### Collections

Mosquitoes were collected biweekly from May to September 2007, from natural and artificial resting sites, by vacuum collection with a portable backpack aspirator as previously described [38]. Briefly, light traps ran from dusk to dawn and were positioned approximately 2 m above ground. Vacuum collections were made twice a week from resting boxes and natural resting sites during this same time period. These collections complemented those from light traps and allowed sampling of mosquitoes in different physiological/behavioral conditions, i.e., nulliparous/parous host-seeking mosquitoes in light traps versus blood-engorged or gravid ones in resting boxes, or allowed the collection of species not attracted to light. Live material was returned to the laboratory, sorted, identified using a chill table and binocular microscope, and frozen at -70°C [39,40]. Point counts were used to estimate bird densities at the study site as previously described [21]. Point counts lasted three minutes, and all birds seen or heard within 100 m of the observed sites during the three-minute counts were recorded. Bird counts were conducted using trained, competent observers. Birds were surveyed in a grid of 110 points, separated by 250 m within a 1.7 km radius from the center of the study site. The grid points were selected, systematically based on sampled strategies generated from previous research [21]. Counts lasted three minutes and were conducted from June 30 through July 29, from 0500 until 1100 local time, with all birds seen or heard within 100 m from the observer recorded.

### Remote sensing data

QuickBird data http://www.digitalglobe.com encompassing the visible and near infra-red (NIR) bands was acquired on July 15, 2008 for the study site. QuickBird multispectral products provided four discrete non-overlapping spectral bands covering a range from 0.45 to 0.72 μm, with an 11-bit collected information depth. The spatial resolution of the data was 0.61m. The clearest, cloud-free imagery available of the contiguous sub-areas of the study site was used to identify mosquito and wild bird sampling sites.

### Base mapping

Base maps of major roads and hydrological networks were created using ArcInfo 9.2^® ^(Environmental Systems Research Institute, Redlands, California) from differentially corrected global positioning system (DGPS) ground coordinates. In this research, fixed surveillance sites were geocoded using a CSI-Wireless (DGPS) Max receiver with a real-time Omni Star L-Band satellite signal, which has a positional accuracy of 0.179 m (+/0.392 m) [31]. A 10m × 10 m grid-based matrix was overlaid on the base maps of the study site, in ArcInfo 9.2^® ^to generate efficient spatial sampling units. A unique identifier was placed in each grid cell. For remote identification of arboviral mosquito and avian habitats, the first step is often to construct a discrete tessellation of the region [41-48].

### Regression analyses

A linear regression, with statistical significance, was determined by a 95% confidence level and used to ascertain whether the proportions of sampled mosquito data differed by grid cell. The linear regression model assumed a random sample between *Y*_*i*_, (sampled mosquito habitat count data), the regress and regressors *X*_*i*1_, ... *X*_*ip*_. A disturbance term *ε*_i_, which was a random variable, was added to this assumed relationship to capture the influence of all habitat parameters sampled on *Y*_*i *_other than *X*_*i*1_, ... *X*_*ip*_. The random error term, *ε*, in a regression analysis of field and remote-sampled *Culex *aquatic model, is typically assumed to be normally distributed with mean zero and variance σ2 [31]. Statistical characteristics of the sampled data were examined in PROC UNIVARIATE. The PLOT option in the PROC UNIVARAITE statement generated histograms and boxplots. The NORMAL option was used to test whether the field and remote-sampled parameters had a normal distribution. The regression analyses was performed using PROC REG. The multiple linear regression model was:

It was important to distinguish the model in terms of random variables and the observed values of the random variables. Thus, we determined *p *+ 1 parameters *β*_0_, ..., *β*_*p*_. In order to estimate the sampled mosquito aquatic habitat parameters, it was useful to use the matrix notation *Y *= *Xβ *+ *ε*, where *Y *was a column vector that included the mosquito count values of *Y*_1_, ..., *Y*_*n*_, which included the unobserved stochastic components *ε*_1_, ..., *ε*_*n *_and the matrix *X*. This matrix was the observed mosquito aquatic habitat parameter values of the regressors expressed as:

In this research, *X *included a column that did not vary across the sampled mosquito data, which was used to represent the intercept term *β*_0_.

The ecological-sampled data was log-transformed before analyses to normalize the distribution and minimize standard error. Multicollinearity diagnostics from the COLLIN option in SAS^® ^were estimated. Residual-based diagnostics for univariate and multivariate conditional heteroscedastic models, previously constructed from clustering field and remote-sampled mosquito habitat parameter estimates have revealed that errors in variance uncertainty estimation can substantially alter numerical predictions models due to multicollinearity [31]. The SAS COLLIN option produced eigenvalues and condition index, as well as proportions of variances with respect to individual-sampled predictor variables in the model. The conditional index scores indicated no significant multicollinearity with the model. It was hypothesized, however, that serial correlation could be a major source of time-varying heterogeneity. In this research, the Durbin-Watson statistic was used to detect the presence of autocorrelation in the residuals from the regression analysis. The Durbin-Watson can test for first-order serial correlation [49]. Usually, the Durbin-Watson statistic is used to test the null hypothesis *H*_0_:*φ*_1 _= 0 against *H*_1_:*φ*_1 _> 0 [49]. The generalized Durbin-Watson statistic is written as:

where  is a vector of OLS residuals and **A**_*j *_is a (*T *- *j*) × *T *matrix. In this research, the generalized Durbin-Watson statistic DW*j *was rewritten as:

where **Q**'_1_**Q**_1 _= **I**_*T *- *k*_, **Q**'_1_**X **= 0, and *η *= **Q**'_1_**u**.

The marginal probability for the Durbin-Watson statistic was:

where *h *= *η*'(**Q**'_1_**A**'_*j*_**A**_*j*_**Q**_1 _- *c***I**)*η*.

The *p*-value, or the marginal probability, for the generalized Durbin-Watson statistic, was computed by numerical inversion of the characteristic function *ϕ*(*u*) of the quadratic form *h *= *η*'(**Q**'_1_**A**'_*j*_**A**_*j*_**Q**_1 _- *c***I**)*η*. The trapezoidal rule approximation to the marginal probability Pr(*h *< 0) was:

where IM[*ϕ*(·)] was part of the characteristic function and E_*I*_(Δ) and E_*T*_(*K*) were integration and truncation errors, respectively. The trapezoidal rule is a way to calculate the definite integral [49]. A numerically efficient algorithm was used to quantify the autocorrelated components in the regression model, which required O(*N*) operations for evaluation of the characteristic function *ϕ*(*u*). The characteristic function was denoted as:

where

By applying the Cholesky decomposition to the complex matrix **V**, we obtained the lower triangular matrix **G **that satisfied **V **= **GG**'. Cholesky decomposition is a decomposition of a symmetric, positive-definite matrix into the product of a lower triangular matrix and its conjugate transpose [49]. The characteristic function was evaluated in O(*N*) operations by using the following formula:

where X* = **G**^-1^**X**.

We tested for serial correlation with lagged dependent variables in the model (Appendix a). When regressors contain lagged dependent variables, the Durbin-Watson statistic (*d*_1_) for the first-order autocorrelation is biased toward 2 and has reduced power [50]. If the Durbin-Watson statistic is substantially less than 2, there is evidence of positive serial correlation [49]. In AUTOREG, two alternative statistics (Durbin *h *and *t*) can be used to test for time varying residuals that are asymptotically equivalent [50]. In this research, we used the *h *statistic, which was written as:

where

, and  was the least squares variance estimate for the coefficient of the lagged dependent variable.

In PROC AUTOREG, an estimation method was used to generate an autoregressive error model using the Yule-Walker (YW) method. The YW method can be considered as generalized least squares using the OLS residuals to estimate the covariances across observation [49]. In this research, we let *φ *represent the vector of autoregressive parameters, *φ *= (*φ*_1_, *φ*_2_,..., *φ*_*m*_)', and we let the variance matrix of the error vector be **ν **= (ν_1_, ..., ν_*N*_)' be **Σ**, *E*(νν' = Σ = σ^2^**V**. If the vector of autoregressive parameters ***φ ***is known, the matrix **V **can be computed from the autoregressive parameters; **Σ **is then σ^2^**V **[49]. Given **Σ**, the efficient estimates of regression parameters *β *were computed using generalized least squares (GLS). The GLS estimates then yielded the unbiased estimate of the variance σ^2^.

The YW method alternated estimation of *β *using generalized least squares with estimation of ***φ***, which the YW equations applied to the sample autocorrelation function. The YW method started by forming the OLS estimate of *β*. Next, ***φ ***was estimated from the sample autocorrelation function of the OLS residuals by using the YW equations. Then **V **was estimated from ***φ***, and **Σ **was generated from **V **and the OLS parameters of σ^2^. The autocorrelation corrected estimates of the regression parameters, *β*, were then computed using GLS and the estimated matrix. The YW equations, solved to obtain  and a preliminary estimate of σ^2^, were **R***ϕ *= -**r**. In this research, we used the equation **r **= (*r*_1 _..., *r*_m_)', when *r*_i _was the lag ***i ***sample autocorrelation. The matrix **R **was the Toeplitz matrix, whose ***i, j***th element was *r*_|*i*-*j*|_. Toeplitz matrix is a matrix in which each descending diagonal from left to right is constant [49]. We specified a subset model. Only the rows and columns of **R **and **r **corresponding to the subset of lags specified were used. The BACKSTEP option was specified for purposes of significance testing. The matrix [**Rr**] was treated as a sum-of-squares-and-cross products matrix arising from a simple regression with *N *- *k *observations, where ***k ***was the number of estimated *Cx. erraticus *habitat parameters in the model.

### Digital elevation model

A three-dimensional model of the study area was constructed based on DEM statistics generated using ArcScene extension of ArcGIS^®^. The DEM used in this research was a raster representation of a continuous surface, originating from the Shuttle Radar Topography Mission (SRTM) which had a spatial resolution of 92 m. The probability distribution of the soil moisture deficit, i.e., statistics of topography, was generated from the DEM data by using a multidirectional flow routing algorithm. The purpose of DEM construction was to extract topographic parameters that may have been associated with the field and remote-sampled EEEV mosquito and bird covariates. A flow apportioning algorithm can delineate a realistic channel network for quantifying hydrogeomorphic properties of simulated drainage patterns using DEMs for identifying floodwater mosquitoes [35].

### Spatial analyses

Kriging models were generated using all sampled abundance count data in Geostatistical Analyst Extension of ArcGIS 9.2^®^. However, based on the evidence that *Cx. erraticus *is likely the primary bridge vector of EEEV in Tuskegee [10] and was the most abundant species sampled in the study site, it was selected for the independent kriging analyses (Table 1). Also, kriging analyses were run for total abundance counts of the sampled bird data in Tuskegee, in 2007. From preliminary data analyses, it was determined that Northern Cardinals were the most abundant avian species in the study site (Table 2). Therefore, a kriged model was generated using the Northern Cardinal data sample points. All the models were created in the ArcGIS 9.3^® ^Geostatistical Analyst Extension.

**Table 1:** Adult mosquito counts for the Tuskegee study site

Mosquito species	Adult counts
*Cx. erraticus*	1,848
*An. crucians*	808
*Cx. territans*	632
*An. quadrimacluatus*	444
*Cx. peccator*	199
*Ae. vexans*	193
*Cq. perturbans*	134
*An. punctipennis*	126
*Ur. sapphirina*	124
*Cx. quinquefasciatas*	82
*Cx. restuans*	51
*Cx. salinarius*	45
*Cs. melanura*	33
*Oc. canadensis*	26
*Oc. spp*	14
*Cx. nigripalpus*	3
*Oc. sollicitans*	1
*Oc. sticticus*	1
*Oc. triseriatus*	1
*Or. signifera*	1
*Ps. columbiae*	1
*Ps. ferox*	1
*An. barberi*	1

**Table 2:** Bird counts for the Tuskegee study site

Species	Abundance (% of count)
Northern cardinal	119 (37.6)
Carolina wren	77 (20.5)
Red-eyed vireo	51 (8.2)
Indigo bunting	50 (8.0)
Tufted titmouse	36 (5.8)
White-eyed vireo	35 (5.6)
Acadian flycatcher	31 (5.0)
Red-bellied woodpecker	14 (2.3)
American crow	14 (2.3)
Blue jay	13 (2.1)
Carolina chickadee	10 (1.6)
Northern parula	9 (1.4)

Spatial linear prediction was performed using ordinary kriging. Geostatistical techniques were used to interpolate the values *Z*(*x*_0_), at a sampled mosquito or bird habitat *Z*(*x*), for unobserved sampling sites *x*_0 _and z_i _= Z(x_i_), i = 1... *n*, using data sampled at nearby sampled habitat locations (x_1_,...x_n_). The kriged-based algorithm computed the best linear unbiased estimator, Ž(x_o_) of *Z*(*x*_0_), for the sampled habitat data, based on a stochastic model of the spatial dependence quantified by the variogram γ(*x*, *y*), by expectation μ(*x*) = *E*[*Z*(*x*)], and by the covariance function *c*(*x*, *y*) of the random field. In this research, the kriging estimator was given by a linear combination:(2.1)

for analyzing the sampled data; where, *z*_*i *_= *Z*(*x*_*i*_) was the weights while w_i _(x_o_) and *i *= 1... *n *was the variance used to minimize any biased condition [35]. The dependent variables were the sampled adult count of mosquitoes or bird data, which were transformed to fulfill the diagnostic normality test prior to performing the kriging. The kriging weights were then used to fulfill the unbiasedness condition in the spatial interpolation of the ecological-dependent variables using:(2.2)

which was given by the ordinary kriging equation system:

The additional parameter μ was a Lagrange multiplier used in the minimization of the kriging error  to honor the unbiased condition in the ecological dataset [51]. The ordinary kriging was given by:

and the interpolation was given by:

with the error variables quantified using:

The semivariogram generated described the spatial dependence, between the sampled mosquito and bird parameters, as a function of the distance between the sampling sites. The semivariogram allowed for mosquito or bird abundance estimations at any point in the study site. The value of prevalence, *Z*, at the coordinate (*x*_*0*_, *y*_*0*_) was estimated from the *n *nearest sampling values:

by the linear formula:(2.3)

The α_*i *_were found by the Lagrange multiplier *λ *and solving the system:

under the constraint

where h_i, j _denoted the distance between any two mosquito or bird sampled locations, located at (x_i_, y_i_), and (x_j_, y_j_), and h_j,0 _was the distance between the two mosquito or bird sampled sites (x_0_, y_0_). The semivariance was defined as *γ *(h) [50-53]. The magnitude of the semivariance in this research was dependent on the distance between sampled mosquito or bird sites. Semivariance of the deviance residuals of the mosquito and bird count data was calculated, and a variogram was constructed to determine if there was evidence of latent spatial autocorrelation in the sampled data. The plot of the semivariances as a function of distance from a point is referred to as a semivariogram [53]. The empirical semivariogram and covariance can provide information on autocorrelation components in ecological-sampled datasets [49].

In this research, parameters of a fitted mathematical function (i.e., the variogram model) included generating a range, a nugget and a sill. The range is the distance at which curve levels of a constant value of semivariance which can indicate the spatial scale of a pattern in an image [49]. The range, or active lag distance, is also the approximate distance at which spatial autocorrelation between sampled data point pairs ceases, or becomes much more variable [54,55]. The value at which the model attains the range, (i.e., the value on the y-axis) is called the sill, while the nugget is usually assumed to be non-spatial variation due to measurement error and variations in the data that relate to shorter ranges than the minimum sampled data spacing [49]. In this research, the sill indicated that the semivariance values had been reached (i.e., the value of maximum variance was equivalent to the variance of the image pixel value), while a non-zero intercept value (i.e., nugget variance) of the varigram model was indicative of the variability of the field and remote-sampled *Cx. erraticus *and Northern Cardinal data quantified at a resolution smaller than the image resolution. A simple quantitative measure of the interpolation performed was determined by generating root-mean square error (RMSE) values for the models. Optimizing the RMSE by minimizing the spatial structure in a *Culex *aquatic habitat model, can generate a pure nugget variogram, of which the level of nugget variance can represent noise characteristics in field and remote-sampled explanatory variables [31]. Additionally, a neighborhood distance search radius provided the mean standard errors of the interpolated values. Interpolation accuracy can be measured by the natural logarithm of the mean squared interpolation error, which can reveal all main effects of parameter estimates, in an autoregressive model, while quantifying several covariate interaction terms [56-59].

## Results

The regression models were able to classify sampled high and low abundance count habitats. Temperature had a significant association with *Cx. erraticus *adult abundance (p < 0.0002). The predictor variable precipitation also presented a significant relationship (p < 0.05). In this research, Durbin-Watson statistics were generated using the AUTOREG procedure in SAS^® ^to estimate whether the OLS regression estimates indicated significant serial correlation with an estimated order of a lagged covariance of 1. The AUTOREG procedure corrected for serial correlation using the YW method. The Durbin-Watson statistic indicated that serial correlation was not significant in the YW corrected model. The YW estimates for the model indicated a R^2 ^= 0.632, F statistics of 39.177, and Durbin-Watson score of 1.935.

**Figure 1:**
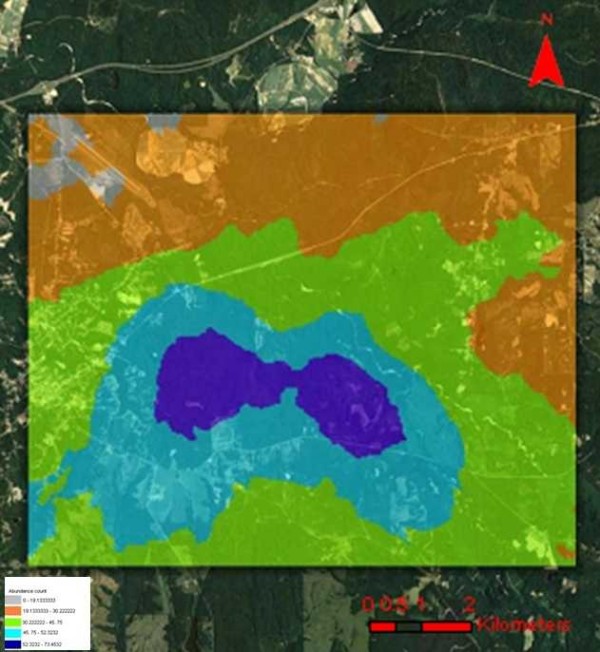
**Predicted *Culex erracticus *abundance data using ordinary kriged model overlaid on a QuickBird visible and near infra-red (NIR) data of the Tuskegee study site**.

**Figure 2:**
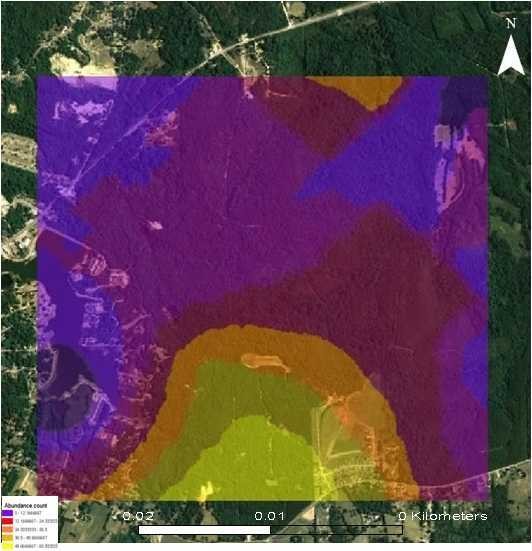
**Predicted Northern Cardinal abundance count data in the Tuskegee study site using an Ordinary kriging algorithm**.

For total mosquito count data, a first-order trend ordinary kriging process was fitted to the semivariogram at a partial sill of 5.041 km, nugget of 6.325 km, lag size of 7.076 km, and range of 31.43 km, using 12 lags. For total adult *Cx. erracticus *count, a first-order trend ordinary kriging process was fitted to the semivariogram at a partial sill of 5.764 km, nugget of 6.114 km, lag size of 7.472 km, and range of 32.62 km, using 12 lags (Figure 1). For the total bird count data, a first-order trend ordinary kriging process was fitted to the semivariogram at a partial sill of 4.998 km, nugget of 5.413 km, lag size of 7.549 km, and range of 35.27 km, using 12 lags. For the Northern Cardinal count data, a first-order trend ordinary kriging process was fitted to the semivariogram at a partial sill of 6.387 km, nugget of 5.935 km, lag size of 8.549 km, and a range of 41.38 km, using 12 lags (Figure 2). To evaluate the accuracy of the models, predictive mean standard error distributions were generated, which revealed that all models were within normal statistical limitations (Table 3).

**Table 3:** Residual model outputs from ordinary kriged models using mean error and root mean square error for the sampled mosquito and bird and count data in the Tuskegee study site.

Data	Ordinary kriging mean error	Ordinary kriging root mean square error
Total bird counts	0.055	1.821

Northern cardinal	0.163	1,642

Total mosquito counts	-0.132	4.664

*Cx. erraticus *count	-4.814	8.535

**Figure 3:**
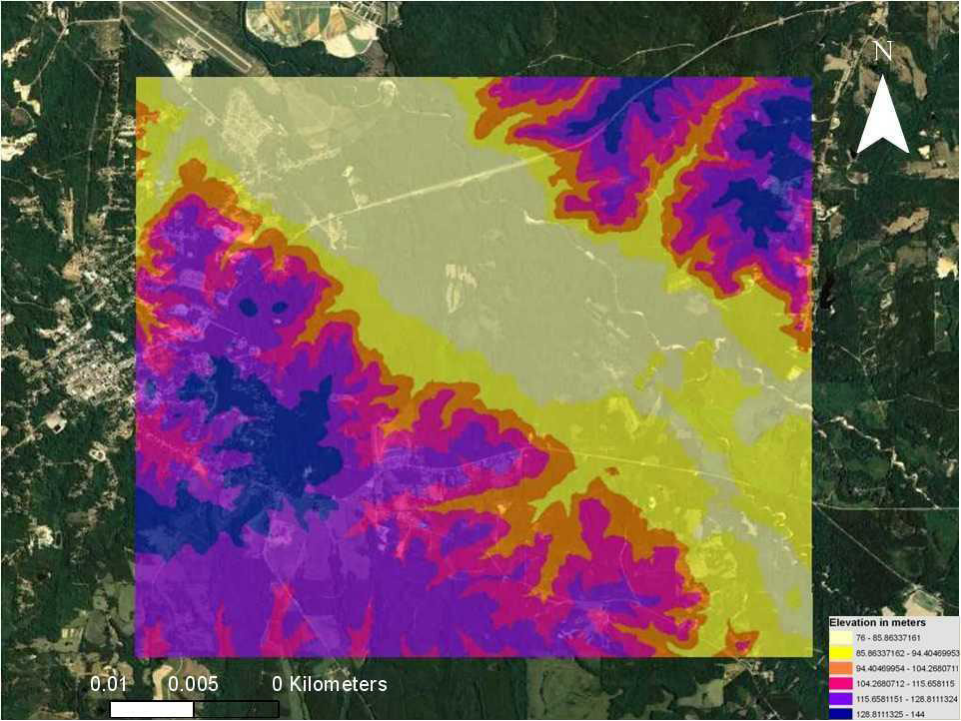
**Digital Elevation Model (DEM) of the Tuskegee study site**.

A DEM of the study site was generated in ArcGIS^® ^(Figure 3). The minimum and maximum range of the elevation in the DEM models were calculated. Pearson's correlation was used to evaluate the linear relationship between mosquito and bird count data and the sampled predictor variable elevation using the SRTM DEM. Results of the DEM analyses indicated a statistically significant inverse linear relationship between total sampled mosquito data and elevation in meters (m) (R^2 ^= -.426; p < .0001), with a standard deviation (SD) of 104.6. The range of the elevation in the DEM had a minimum value of 0 m, with a maximum value of 431 m. The results of the total sampled bird data and elevation were (R^2 ^= -.511; p < .0001), with a SD of 22.97. The range of the elevation in the DEM had a minimum value of 0 m, with a maximum value of 439 m. DEM statistics also indicated a significant inverse linear relationship between total sampled *Cx. erracticus *data and elevation (R^2 ^= -.471; p < .0001), with a SD = 111.6. The range of the elevation in the DEM had a minimum value of 0 m, with a maximum value of 487 m. The results of the total sampled Northern Cardinal data and elevation was (R^2 ^= -.583; p < .0001), with a SD = 114.2. The range of the elevation in the DEM had a minimum value of 0 m, with a maximum value of 501 m (Table 4).

**Table 4:** Pearson correlation for mosquito and bird sampled data and the sampled predictor variable elevation in the Tuskegee study site.

Predictor variables	Statistical tests	Significance level	Elevation (m)
Total mosquito count data	Pearson Correlation	1	-.426
	Sig. (2-tailed)	<.0001	<.0001
	N	141	118

Total bird count data	Pearson Correlation	1	-.511
	Sig. (2-tailed)	<.0001	<.0001
	N	141	118

*Cx. erraticus *data	Pearson Correlation	1	-.471
	Sig. (2-tailed)	<.0001	<.0001
	N	141	118

Northern cardinal data	Pearson Correlation	1	-.583
	Sig. (2-tailed)	<.0001	<.0001
	N	141	118

## Discussion

*Culex erraticus *was the most abundant mosquito species collected during this study in central Alabama bottomland freshwater wetlands, which was ~6 km from the center of Tuskegee and ~1.5 km east of a populated area north of highways US-29/AL-81. This species previously yielded the highest number of EEEV-infected pools in Tuskegee [21]. Habitat requirements of *Cx. erraticus *are shallow water [60-66], especially overgrown with surface plants or grassy margins, such as streams, lakes or impoundments [18]. This species may be collected in high numbers during hot weather, and even during drought, periods in July and August [10]. Bloodfeeding hosts of *Cx. erraticus *include: birds (27-70%), mammals (23-67%), and reptiles (2-20%), suggesting great host flexibility based on relative availability of hosts [16].

The bird communities present in the Tuskegee study site are typical of reforested areas of bottomland hardwood [10]. The level of vector contact with different bird species in a given area is essential in identifying those avian species that are most likely to serve as important amplifiers for arboviral enzootics [15]. Birds that frequent edge habitats are assumed most likely to transmit EEEV between localized pest mosquitoes and humans or horses [21]. Woodland and swamp birds species captured within an EEEV focus in Michigan had high antibody prevalence, but seroconversion of EEEV to other urban bird species in open areas increased during peak transmission to horses [65].

Previous research of EEEV in Alabama also has suggested the importance of hatch-year birds as catalysts for epidemic transmission. For example, wild birds involved in EEEV transmission in Alabama forested areas included: Yellow-Crowned Night-Heron, Carolina Chickadee, Northern Mockingbird and Great Blue Heron [21]; in Florida, the species were: Bluejay, Northern Mockingbird, Rufus-Sided Towhee, Loggerhead Shike, Northern Cardinal, Cattle Egret and the gamebirds Pheasant and Chukar Partridge [8]; and, in Louisiana, the species were: White-Throated Sparrow, Northern Cardinal, House Sparrow, Rufus-Sided Towhee, Carolina Chickadee and Yellow-Rumped Warbler [19]. Similarly, the House Sparrow is a primary vector for WNV, EEEV and SLE in Louisiana [17]. Most of these listed birds are included on the Audubon Society checklist as very common species to Alabama for spring, summer and fall. Notably, birds involved in EEEV tend to be less urban than WNV/SLE avian hosts, though considerable host species overlap is apparent between diseases and between geographic regions [16]. Chickadees, Northern Cardinals, Tufted Titmice, Blue Jays, American Crows, Brown-Headed Cowbirds and Red-Billed Woodpeckers inhabit both wooded and human-populated areas [62]. American Crows may forage in wooded locations but roost at night in urban and suburban areas [8,46].

*Culex erraticus *was the most common mosquito collected at the study site. Because of its density, it is likely that *Cx. erraticus *plays a major role in perpetuating EEEV transmission. *Cx. erraticus *is a member of the subgenus *Melanoconion*, a largely tropical group of mosquitoes. *Cx. erraticus *is the most common member of that subgenous in the United States and is distributed throughout the eastern and the upper midwest and westward to California [16]. Since *Cx. erraticus *is a neotropical species which plays a major role in the transmission of EEEV in Tuskegee, the geographical distribution of EEEV in Alabama might resemble that seen in EEEV foci in tropical areas (e.g., South America). The probability that a mosquito will feed on a reservoir host is one of the most influential variables affecting the geographical distribution of an arthropod mosquito vector [8]; therefore, determining spatial patterns of *Cx. erraticus *host preferences can play a significant role in controlling the development of avian enzootics of EEEV in the Tuskegee study site.

Temperature and precipitation were important predictor variables in the regression model. Environmental conditions such as temperature, and precipitation, have an important effect on the distribution of the mosquitoes that harbor arboviruses, thereby, influencing seasonal virus activity [3,10,11]. Previous regression analysis estimates derived from multiple field and remote-sampled predictor variables, collected from nine sites within Cook County, Illinois, revealed that adult *Culex *population was positively associated with temperature [57]. The model output indicated that precipitation was negatively associated to mosquito abundance in 2002, 2003 and 2005 (P <0.05), but positively associated in 2004 (P <0.05). Naturally occurring outbreaks of EEEV are usually observed during periods of hot, rainy weather [3]. These weather conditions are ideal for expansion of *Cs. melanura *and other mosquito populations [63]. Outbreaks of EEEV in horses and humans are expected to occur from midsummer to late summer, with August being the peak month of incident cases in much of the United States [11]. An evaluation of EEEV in horses in Michigan, during five outbreaks between years 1972 and 1991, revealed an increase in *Cs. melanura*, *Cq. perturbans*, and *Ae. vexans *as vectors, wild and domestic birds as reservoir hosts, humans and commercial poultry flocks as incidental hosts, with an increase in the state-wide annual precipitation and an increase in region-specific precipitation [19]. Temperature and precipitation is likely involved in early season enzootic transmission and late season epizootic amplification of the EEEV in wild bird populations at the Tuskegee study site. It is possible that *Cx. erraticus *may become important as a bridge vector of EEEV in the southeastern United States, as human populations continue to move closer to sylvatic sites, where populations of this mosquito have access to avian reservoirs and during specific time frames throughout the year, when there is fluctuation in meteorological variables. An important variable in the amplification from the enzootic cycle of arboviral encephalitides is the degree of contact between avian hosts and mosquito vectors [21]. *Culex erraticus *has a minimally infection rate of 3.2, from mid-June to mid-September [16].

In this research, there was reasonable overlap between the kriged predictive maps. Much of this overlap was related to the location of the Tuskegee National Forest ponds, which were near the center of the sampling grid and composed of forested land cover. High mosquito abundance have been found in land cover sites classified as having a low-to-moderate range of built environment and high forest composition [57]. This data suggests that birds and mosquitoes frequently forage several kilometers beyond their preferred habitats. Mosquito species differ in their overall preference for different classes of host (e.g., mammals versus birds versus reptiles), in the times of day that they are most active in seeking bloodmeals, and the heights at which they forage [21]. Many of the mosquito hosts sampled were edge species, birds that are most common at the interface between forested and disturbed landscapes, such as cardinals, corvids, and parids. For example, Northern Cardinals were previously found six times more abundant in habitats surrounded by open space [11].

The semivariograms, generated in ArcGIS Spatial Analyst, modeled the structure of spatial variability in the field and remote-sampled *Cx. erracticus *and Northern Cardinal habitat data. The semiovariograms were used to fit models of the spatio-temporal correlation of the sampled mosquito and bird parameters. The semivariogram and covariance functions quantified the nearby habitats as a measure of the strength of statistical correlation and as a function of distance between sampled habitats. Linear predictors were generated by incorporating models of the covariance of the random function using a weighted moving average interpolation. The semivariogram of residuals from the regression models generated from the sampled covariates, with stationary errors, were used to estimate the covariance structure of the underlying spatial structural processes in the data.

For prediction kriging, the bias of the semivariogram estimates induced, by using residuals instead of errors, has only a minor effect, as the bias is small for small lags [49]. However, in this research, error in the spatial covariance patterns generated from the estimated regression coefficients may have been quite substantial due to the excess land cover heterogeneity between the sampled habitats. To spatially analyze a mosquito vector, one must understand that insect populations are typically heterogeneous in their spatial densities, responding to multivariate habitat characteristics and environmental controls [25]. Additionally, kriging may not be widely used for predicting EEEV distribution at a local scale, because of the common finding of non-stationarity in ecological-sampled data. The violation of the stationarity assumption may affect the validity of kriged surfaces, since a common metric derived from the semivariogram is not enough to capture spatial variations in EEEV parameters observed at a local scale. The process of using kriged-based algorithms for mosquito and bird parameters may require estimation of the best model parameters, and an assessment of the resulting model accuracy, before it can be used as a predictive tool for evaluating EEEV mosquito and bird habitat data. Diagnostic checking error residuals in an EEEV model may enable intervention efforts spatially targeting productive mosquito and bird habitats based on field and remote-sampled data, by using the asymptotic distribution of parameter estimates from a residual autocovariance matrix.

The distribution of Northern Cardinals was interesting, as this species was by far the most abundant, prevalent, and evenly distributed bird species detected in the Tuskegee study site. This species has also expanded range over the last century due to mild winter patterns and increased use of bird feeders for winter survival [11]. Despite the very high abundance of Northern Cardinals, this species appeared only moderately attractive to host-seeking mosquitoes [10]. Northern Cardinals are common in both forest and urban habitats [19] Radio tracking data suggests that individual cardinals are highly territorial of small plots of land and do not typically migrate more than 50 m from their roosts [14]. It is possible that the high abundance and prevalence of Northern Cardinals prevented smaller passerine EEEV hosts from reaching higher densities. The spatial patterns of total bird counts, Northern Cardinal counts, did not significantly differ; whereas, the spatial pattern of *Cx. erraticus *significantly differed from the overall mosquito counts.

Broad-scale quantification of topography, using the spatial hydrological model of the study site, visually discriminated specific land cover features associated with the mosquito and bird-sampled data with good accuracy. The DEM captured all hydrologic characteristics, determining the flow paths of streams, e.g., watershed boundaries in the study site. Elevation was found to be significantly associated with the sampled mosquito and bird data in the study site. Elevation is directly related to temperature, which effects mosquito survivorship [35]. Because many birds rest in trees, it can be argued that mosquitoes will search for bloodmeals in trees, where their avian hosts would be found. Therefore, mosquito preference for bird hosts influences their attraction to higher elevation. Frequent blood feeding can occur by *Culex *mosquitoes on abundant urban passerine birds [35]. Feeding, primarily on EEEV-competent avian hosts during the amplification period of the epidemiological cycle, maximizes the intensity of the epidemic in mosquitoes [64].

Modeling field and remote-sampled explanatory variables of EEEV in newer GIS offer an attractive and better alternative to traditional disease mapping approach. Such an approach lends itself to the development of powerful predictor models for estimating multiple environmental-sampled variables of EEEV mosquito and avian sampling sites. Newer GIS software packages provide a wide range of tools for data analysis, using cartographic modeling for identifying mosquito and bird data and estimating spatial dependency in the sampled ecological datasets. For example, a GIS-based model can generate sampling prediction error distributions that are well defined for identifying explanatory variables associated with prolific mosquito and avian habitats based on sampled count data. Therefore, GIS/remote sensing maps of mosquito and bird data associated with EEEV have a direct use in public health programs and targeted interventions. Monthly risk maps, showing the relative danger of regional EEEV transmission, based on mosquito and bird abundance data, can be constructed. Risk maps can then be updated weekly as epidemic triggers are identified and quantified, enabling GIS/remote sensing-based spatial predictions of favorable future conditions for EEEV transmission [25].

Additionally, a graduated, systematic GIS sampling methodology can adjust for sampled ecological covariates, a technique that can identify more georeferenced *Cx. erraticus *and wild birds habitat clustering sites within urban environments than random sampling strategies. A major advantage of using GIS-based models is that the sampling prediction error distributions are well defined for identifying explanatory variables associated with prolific mosquito and avian habitats based on sampled count data. Therefore, designing and developing control strategies, based on GIS/remote sensing data, and models can provide effective entomological tools to reduce mosquito arboviral vectors in conjunction with high density foci by identifying critical features of landscape for locating productive areas in a study site. Since it is more feasible to expand surveys to targeted habitats, based on spatially selected potential foci [31], a systematic GIS surveillance sampling frame, using QuickBird data and geostatistical predictive algorithms, can focus on specific habitats, which can allow for intensified entomological surveillance at specific habitats, while not increasing overall sampling efforts. Random interventions are excessive and wasteful, as arboviral vectors are not themselves randomly distributed [25] and spatio-temporal sampled abundance counts of mosquito and bird habitats fluctuate constantly [37].

Substantive variations in mosquito and bird abundance, in relation to arboviral infections, pose a challenge for surveillance programs; yet, spatial statistics and GIS surveillance sampling strategies have not been applied in correspondence to these changes in Alabama. For example, in 2007, 24 human cases of WNV were reported to CDC ArboNET, 13 of which were in Montgomery County; however, mosquito sampling has been limited to only the north quadrant of Alabama in recent years [67]. Bird data also were collected for every county in Alabama in 2002-2003 [68]; however, reporting has since stopped in most counties. Although an epidemic of SLE cases in humans occurred in 1975 in Birmingham, Alabama (32 cases) [23], which led to formation of a SLE mosquito surveillance program, this program no longer exists and should be replaced. The wide geographical range of the 1975 epidemic [69] highlights critical need for interdisciplinary surveillance in Alabama and other southeastern states. Similar epidemics in Arkansas (1991) and Louisiana (2002) revealed *Culex quinquefasciatus *as the primary vector of SLE and/or WNV [70]. The high seroprevalence (36%) of primates exposed outdoors during the 2002 WNV epidemic in Louisiana suggested that human exposure risks are also likely high[17]. Therefore, endemic pathogens for EEEV, WNV and SLE can be target systems used to develop surveillance models that incorporate predictive algorithms; as well as field-sampled and remotely sensed data.

The surveillance system described in this paper could also be incorporated to develop strategies for the detection of avian influenza. The early warning signs (i.e. interaction of vector-host-virus) suggest a valid concern that Alabama is unprepared in the event of an avian flu pandemic. Low pathogenicity H5N1 strains have been detected in wild bird migratory populations in the United States [67]. Potential routes for introduction of the H5N1 virus into Alabama include migration of infected wild birds. Recent trends suggest that H1N1 can now remain virulent in ducks for longer durations, which may allow water fowl to shed the virus as they migrate through areas of outbreaks [71]. Reports of 87 positives for H1N1 from carcasses of 19 avian species in Sweden and Denmark suggest that avian influenza is on the move [72].

Furthermore, replication of the GIS sampling techniques, and the statistical algorithms used in this research, can provide detailed distributions for targeting highly productive mosquito habitats in other geographic areas outside of the United States. For example, the models generated in this research can be used to control other encephalitis-type, enzootic arboviral diseases, such as Venezuelan equine encephalomyelitis virus (VEE), which is transmitted by mosquitoes endemic in Central American and South American countries [73]. VEE is caused by encephalitic alphaviruses in the family *Togaviridae *similar to EEEV. Encephalitic alphaviruses have caused repeated epidemics and equine epizootics since the 1920s. For example, a major outbreak of VEE in Venezuela and Colombia, during 1995, involved an estimated 100,000 people [73]. Therefore, quantifying enzootic foci of VEE surveyed in unknown sites in a particular geographic region (e.g., Mexico), using multi-temporal sampled QuickBird data in GIS, coupled with ground surveillance data, can be essential for designing pathogenesis studies, simulating natural infection of vertebrates. Characterization of various statistical techniques can then be applied to the sampled data to identify high density habitats in determined areas, where environmental-sampled predictors may not be the only factor influencing productivity. Post-classification can include validating VEE mosquito parameters using extensive ground truthing to identify regions of highly prolific habitats.

In conclusion, regression models revealed that temperature and precipitation had significant association with sampled *Cx*. *erraticus *adult abundance count data. Kriging techniques developed a spatial linear prediction model of potential mosquito and bird habitats for the Tuskegee study site. The application of kriging reduced constraint on the interpolated value of the sampled mosquito and bird abundance data, to take advantage of distance and direction in the interpolation process and to minimize the variance of unexpected error. Topographic descriptors, derived from the DEM, supported a quantitative analysis of the spatial distribution and configuration of the georeferenced mosquito and bird sampled data in the study site. Mosquito indicators combined with other environmental information such as temperature and precipitation, wild bird population, and EEEV strains, may offer more precise evaluation of human EEEV disease risks. Continued development of spatio-temporal models, using GIS, remote sensing data and spatial statistics, can estimate vector and infected host distribution, which can further predict the distribution of EEEV based on ecological-sampled covariates.

## Competing interests

The authors declare that they have no competing interests.

## Authors' contributions

BJ conceived the study and led the drafting of this manuscript; JL and SP contributed to the interpretation and results of the remote models. NB supervised the mosquito data collection; GH supervised the bird data collection; CM, LE, EC and RN helped in the entomological analyses of the manuscript; TU is the principal investigator of the study. All authors interpreted the results and wrote the paper.

## Appendix A

### Using the AUTOREG procedure for generating the Generalized Durbin-Watson Tests from the ecological sampled EEEV parameters

Initially we used the regression model: **Y **= **X*β ***+ **ν **where **X **was an *N *× *k *data matrix, *β *was a *k *× I coefficient vector, and **ν **was a *N *× I disturbance vector. The error term **ν **was assumed to be generated by the *j*th-order autoregressive process ν_*I *_= *ε*_*I *_- *φ*_*j*_ν_*I - j *_where |*φ*_*j*_| < I, *ε*_*I *_was a sequence of independent normal error terms, generated from the analyses of the EEEV data with mean 0 and variance σ^2^. We used the Durbin-Watson statistic to test the null hypothesis *H*_0 _: *φ*_1 _= 0 against *H*_1 _: -*φ*_1 _> 0. The generalized Durbin-Watson statistic was:

where  were OLS residuals. We used the matrix notation, 

where **M **= **I**_*N *_- **X**(**X'X**)^-1^**X**' and **A**_*j*_

was a (*N *- *j*) × *N *matrix: 

and there were *j *- I zeros between -I and 1 in each row of matrix **A**_*j*_. The QR factorization of the design matrix yielded a *N *× *N *orthogonal matrix **Q**: **X **= **QR **where R was an *N *× *k *upper triangular matrix. There existed an *N *× (*N *- *k*) sub-matrix of **Q **such that **Q**_1_**Q**'_1 _= **M **and **Q**'_1_**Q**_1 _= **I**_*N *- *k*_. Consequently, the generalized Durbin-Watson statistic was stated as a ratio of two quadratic forms:  where λ_*j*1_... λ_*jn *_were the upper *n *eigenvalues of **MA**'_*j*_**A**_*j*_**M **and ξ_*I *_was a standard normal variate, and *n *= min(*N *- *k*, *N *- *j*). These eigenvalues were obtained by a singular value decomposition of **Q**'_1_**A**'_*j*_. The marginal probability (or *p*-value) for *d*_*j *_given *c*_o _was

where .

When the null hypothesis *H*_o _: *φ*_*j *_= 0 held, the quadratic form *q_j _*had the characteristic function 

The distribution function was uniquely determined by this characteristic function: 

We tested *H*_o _: *φ*_4 _= 0 given *φ*_1 _= *φ*_2 _= *φ*_3 _= 0 against *H*_1 _: -*φ*_4 _> 0, using the marginal probability (*p*-value) and : 

where  and  was the calculated value of the fourth-order Durbin-Watson statistic from the ecological sampled EEEV data.
